# A potential new enriching trial design for selecting non-small-cell lung cancer patients with no predictive biomarker for trials based on both histology and early tumor response: further analysis of a thalidomide trial

**DOI:** 10.1002/cam4.74

**Published:** 2013-04-24

**Authors:** Siow Ming Lee, Allan Hackshaw

**Affiliations:** 1 Department of Oncology, University College London (UCL) Hospitals and UCL Cancer Institute250 Euston Road, London, NW1 2PG, United Kingdom; 2 Cancer Research UK and UCL Cancer Trials Centre, University College London90 Tottenham Court Road, London, W1T 4TJ, United Kingdom

**Keywords:** Antiangiogenics, clinical trials, lung cancer, NSCLC, thalidomide

## Abstract

There are few predictive biomarkers for antiangiogenic trials in lung cancer. We examine a potential treatment strategy in which a patient group is enriched using both histology and an early assessment of response during standard chemotherapy, and where a new agent is given for the remainder of chemotherapy and as maintenance. We performed a retrospective analysis of 722 stage IIIB/IV non-small-cell lung cancer patients from a double-blind placebo-controlled trial of thalidomide or placebo 100–200 mg/day, combined with gemcitabine/carboplatin (for up to four cycles), then given as single agent maintenance therapy. There was a significant statistical interaction between treatment and histology, with a possible benefit among squamous cell cancer (SCC) patients. We examined 150 SCC patients who were “nonprogressors” (stable disease or complete/partial response) after completing the second chemotherapy cycle. Endpoints were progression-free survival (PFS) and overall survival (OS). Among the 150 patients nonprogressors after cycle 2 (thalidomide, *n* = 72; placebo, *n* = 78; baseline characteristics were similar), the hazard ratios (HRs) were: OS = 0.76 (95% CI: 0.54–1.07) and PFS = 0.69 (95% CI: 0.50–0.97). In 57 patients who had a complete/partial response, the HRs were: OS = 0.63 (95% CI: 0.34–1.15) and PFS = 0.50 (95% CI: 0.28–0.88). SCC patients who were nonprogressors after 2 cycles of standard chemotherapy showed evidence of a benefit from thalidomide when taken for the remainder of chemotherapy and as maintenance. This strategy based on histology and, importantly, early assessment of tumor response, as a means of patient enrichment, could be examined in other lung cancer studies. Such an approach might be suitable for trials where there are no predictive biomarkers.

## Introduction

Lung cancer remains difficult to treat, largely because most patients present with advanced disease that is not amenable to surgery or radical radiotherapy. Currently, patients with advanced non-small-cell lung cancer (NSCLC) are frequently selected for treatment based on their tumor histology. In the first-line JMDB trial, survival was significantly improved among patients with nonsquamous histology who received cisplatin/pemetrexed, while cisplatin/gemcitabine was more effective for those with squamous cell cancer (SCC) [1]. Similarly, in the JMEN study of maintenance pemetrexed in advanced NSCLC patients, overall survival (OS) and progression-free survival (PFS) were only improved in nonsquamous patients [2].

In 2009, we reported the results of a large randomized trial of thalidomide (Study 14), an oral antiangiogenic agent, when combined with first-line gemcitabine/carboplatin in advanced stage IIIB/IV NSCLC. There was no overall benefit (hazard ratio [HR] = 1.13, 95% confidence interval [CI]: 0.97–1.32) [3]. However, there was evidence of a differential treatment effect according to histological subtype, and the interaction between histology and thalidomide/placebo was statistically significant (*P* = 0.006). Among patients with SCC, the 2-year survival rates were 20% (thalidomide) versus 12% (placebo): a difference of +8% (95% CI: −1 to +17; *P* = 0.10). The OS Kaplan–Meier treatment curves for squamous patients appeared to overlap early, after which they separated. We believe that the overlap early on could be due to including patients who are unlikely to benefit from chemotherapy, but patients with a tumor response or stable disease might have more opportunity to benefit from an agent like thalidomide, when combined with chemotherapy. We therefore investigated the strategy of using histology and early tumor response together in selecting patients in whom new therapies are more likely to show benefits.

## Materials and Methods

We conducted a further analysis of a randomized double-blind placebo-controlled trial of thalidomide. The study is registered with ISRCTN (77341241). Full details have been described elsewhere [3]. Briefly, the trial included 722 patients with histologically or cytologically confirmed stage IIIB/IV NSCLC (recruited 2003–2005), with no prior chemotherapy or radiotherapy for their cancer. The cytotoxic drugs were gemcitabine 1200 mg/m^2^ given intravenously (days 1 and 8), and carboplatin area under the curve 5 or 6, dependent on method of glomerular filtration rate estimation (day 1); and given for up to four cycles. Patients were randomly allocated to receive thalidomide or matching placebo capsules, to be taken orally once daily from the start of chemotherapy for 2 years. The starting dose was 100 mg/day during chemotherapy and, if tolerated, increased to 150 mg/day at the end of chemotherapy for 1 month, then to 200 mg/day maintenance dose for the rest of the trial.

Assessments were performed at each chemotherapy cycle, which included physical and neurological examinations, hematology and chemistry, a chest radiograph, and (usually after cycles 2 and 4) computed tomography scan of the thorax and abdomen. After chemotherapy, the same assessments were scheduled every 2 months for the first 2 years, then every 3 months.

Statistical analyses, that is, HRs and Kaplan–Meier curves, were examined for two endpoints: OS and PFS. PFS was taken as the date of first recurrence or death. OS and PFS were measured from the date of the tumor assessment after cycle 2, or from the date of randomization. We examined all patients according to histology and whether they achieved disease control to treatment by the end of chemotherapy cycle 2 (i.e., stable disease, or partial or complete response, referred to as “nonprogressors”). Tumor response was assessed using the Response Evaluation Criteria in Solid Tumors [4]. Conducting trials separately in patients with squamous and nonsquamous histology is now commonplace, as are maintenance studies that focus on patients who respond to initial first-line treatment. The analyses we present here are simply a combination of these two aspects.

## Results

Of the 722 patients randomized, 483 (67%) had nonsquamous histology. The remaining 239 (33%) had tumors of SCC, of whom 150 patients had at least stable disease at the end of chemotherapy cycle 2. Among these patients (*n* = 150), 93 (62%) had stable disease, 55 (37%) had a partial response, and 2 (1%) had a complete response.

Baseline characteristics were similar between the thalidomide and placebo groups among the 150 SCC patients who were nonprogressors, that is, had at least stable disease (Table 1), including tumor response.

**Table 1:** Baseline characteristics among patients with squamous histology only who had at least stable disease at the end of chemotherapy cycle 2

	Thalidomide (*N* = 72), *n* (%)	Placebo (*N* = 78), *n* (%)	*P*-value for difference between treatment groups
Age at randomization (years)			
≥50	68 (94.4)	76 (97.4)	0.35
Median	63	66	
Range	36–77	48–83	
Sex			
Male	55 (76.4)	59 (75.6)	0.91
Female	17 (23.6)	19 (24.4)	
ECOG performance status			
0	24 (33.3)	24 (30.8)	0.82
1	39 (54.2)	46 (59.0)	
2	9 (12.5)	8 (10.3)	
Stage			
IIIb	44 (61.1)	48 (61.5)	0.96
IV	28 (38.9)	30 (38.5)	
With pleural effusion (IIIb)	8 of 44	18 of 48	
Tumor response at the end of cycle 2			
Complete response	2 (2.8)	0	0.34
Partial response	23 (31.9)	32 (41.0)	
Stable disease	47 (65.3)	46 (59.0)	

**Figure 1:**
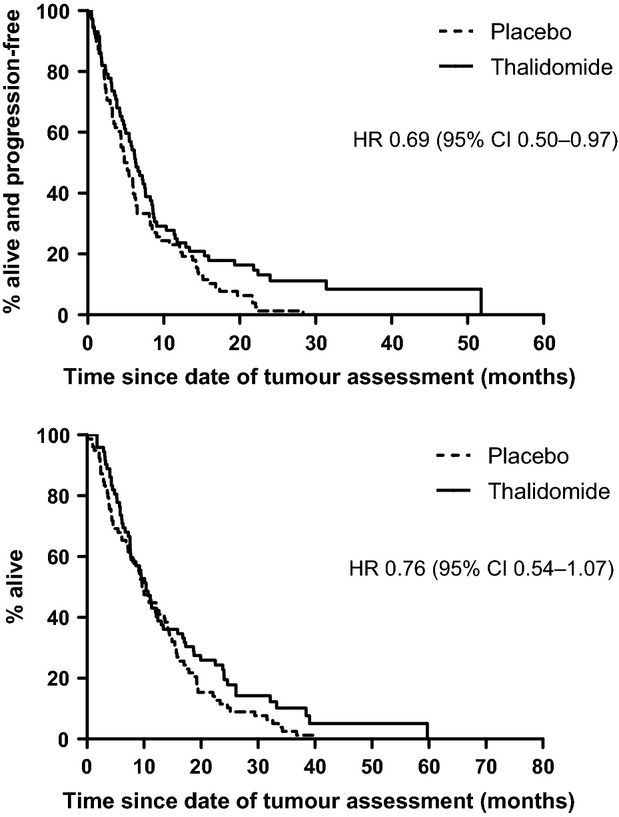
Progression-free survival (upper) and overall survival (lower) among 150 patients with squamous histology who had at least stable disease after chemotherapy cycle 2 (tumor assessment made at the end of cycle 2).

Table 2 shows the results on OS and PFS. There was some evidence of a benefit for both outcomes among all 239 SCC patients (HRs of 0.84 for each endpoint). The effect was greater when focusing only on the *n* = 150 nonprogressors after cycle 2; the PFS HR = 0.71 (95% CI: 0.51–0.99, *P* = 0.04). Figure 1 is the Kaplan–Meier curves for PFS. After adjustment for the baseline characteristics (age, sex, performance status, tumor stage, and presence of pleural effusion), the HR = 0.69 (95% CI: 0.49–0.98, *P* = 0.037). When OS and PFS were measured from the tumor assessment date following cycle 2, the results were: PFS HR = 0.69 (95% CI: 0.50–0.97, *P* = 0.03) and OS HR = 0.76 (95% CI: 0.54–1.07, *P* = 0.12). These became 0.67 (95% CI: 0.48–0.95, *P* = 0.02) and 0.75 (95% CI: 0.53–1.06, *P* = 0.11), after allowing for the baseline characteristics. The lack of statistical significance for some of the comparisons in Table 2 is expected because the trial was not powered for these subgroup analyses.

**Table 2:** Summary results for overall survival and progression-free survival according to histology (the hazard ratio is for thalidomide vs. placebo)

	OS			PFS		
		
	Number of events/Number of patients	HR	*P*-value	Number of events/Number of patients	HR	*P*-value
All patients	665/772	1.13 (0.97–1.32)	0.12	698/722	1.10 (0.95–1.28)	0.20
Squamous cell lung cancer only	218/239	0.84 (0.64–1.09)	0.19	229/239	0.84 (0.64–1.09)	0.19
Squamous patients who were nonprogressors^1^ after two cycles	134/150	0.77 (0.55–1.08)	0.13	143/150	0.71 (0.51–0.99)	0.04
OS and PFS measured from tumor assessment date		0.76 (0.54–1.07)	0.12		0.69 (0.50–0.97)	0.03
Nonsquamous cell lung cancer only	447/483	1.32 (1.10–1.60)	0.004	469/483	1.26 (1.05–1.52)	0.013
Nonsquamous patients who were nonprogressors^1^ after two cycles	268/295	1.40 (1.10–1.78)	0.007	286/295	1.31 (1.04–1.66)	0.02

PFS, progression-free survival; OS, overall survival. OS and PFS measured from date of randomization unless otherwise indicated.

1 Stable disease, partial, or complete response.

**Figure 2:**
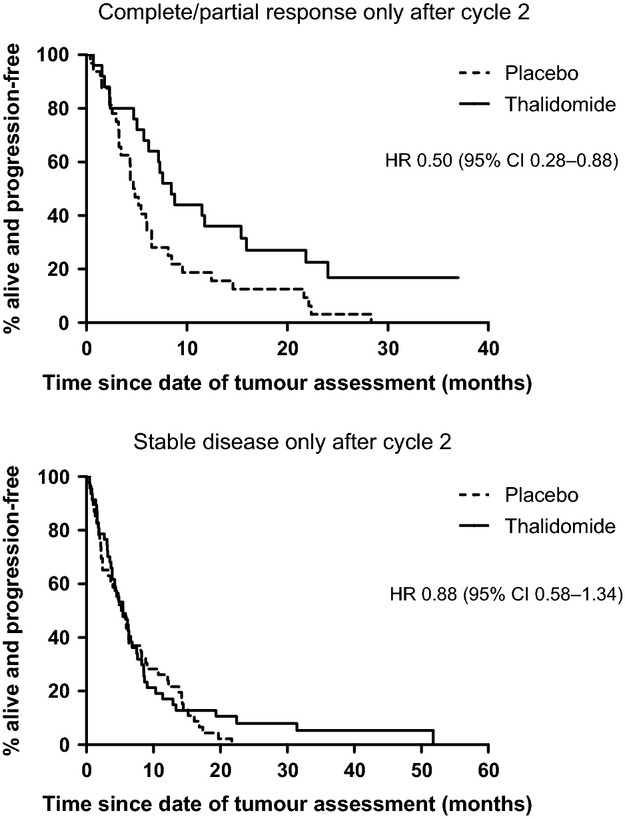
Progression-free survival among 150 patients with squamous histology, according to type of response after cycle 2 (57 with complete/partial response and 93 with stable disease).

Thalidomide seemed to be beneficial in all 150 SCC patients who were nonprogressors, but with a greater effect among the 57 patients who had a complete/partial response only (PFS HR = 0.50, 95% CI: 0.28–0.88, *P* = 0.02 and OS HR = 0.63, 95% CI: 0.34–1.15, *P* = 0.13), than the 93 patients who had stable disease only (PFS HR = 0.88, 95% CI: 0.58–1.34, *P* = 0.54 and OS HR = 0.82, 95% CI: 0.53–1.25, *P* = 0.35; Fig. 2). Although the effect in the patients with stable disease was smaller and not statistically significant, we cannot reliably rule out a potential benefit, due to the smaller sample size.

There was no benefit associated with thalidomide in patients with nonsquamous histology. In fact the data indicated that both OS and PFS were worse in the thalidomide group (Table 2).

Among the 150 SCC patients who were nonprogressors after cycle 2, the percentage with grade 3/4 adverse events were: 51% (thalidomide) versus 49% (placebo) for hematological toxicities and 32% (thalidomide) versus 23% (placebo) for nonhematological toxicities, similar to that found for all trial patients. The main adverse event found in all patients was thrombotic events, and the HR among the 150 patients was 1.29 (95% CI: 0.54–3.12), lower than in all patients (1.74, 95% CI: 1.20–2.52), probably because these events were more likely to occur earlier on [3].

We examined duration of study drug among the 150 patients who had at least stable disease. Ten patients (seven placebo and three thalidomide patients) continued until they died. Among the others, the median time (25th–75th per centiles) from the date of tumor assessment after cycle 2 until they stopped study drug was 6.1 (2.8–12.1) and 3.8 (1.6–8.5) months in the placebo and thalidomide groups, respectively. This difference was just statistically significant (*P* = 0.04), but consistent with data from all patients where the drug duration for thalidomide patients tended to be shorter because of adverse events, such as thrombotic events.

Only four patients received additional chemotherapy: placebo (pemetrexed *n* = 2, docetaxel *n* = 1) and thalidomide (etoposide/cisplatin), and there were only five patients in each trial group who had a biological agent. The proportion who received radiotherapy was also similar: 58% (placebo, 45/78) and 51% (thalidomide, 37/72).

## Discussion

Our analyses suggest that histology combined with tumor response after two cycles of chemotherapy could be used to identify a group of patients in which some antiangiogenics might be more effective. In our particular case, these were patients with SCC who had at least stable disease and treated with thalidomide. Our study is a post hoc analysis, which cannot be used to claim effectiveness of thalidomide, and a prospective study needs to be conducted to confirm or refute our findings. It is possible that this strategy by selecting patients who have at least stable disease after cycle 2, as a means of patient enrichment, could lead to a better prognosis group who are more likely to respond to many other new antiangiogenics and novel agents without predictive biomarkers and not just thalidomide.

We used tumor response after chemotherapy cycle 2 because it is routine to perform tumor assessment at this time, and it also provides a good balance between not excluding too many patients (63% of squamous patients would be included on this basis) and a worthwhile treatment benefit (PFS HR = 0.71), that was statistically significant (*P* = 0.04). If stable disease were also excluded, only 24% of squamous patients would be selected.

Previous trials have used patient or tumor characteristics to preselect patients but only to evaluate a new treatment when given as maintenance (i.e., the assessment of tumor response is made *after* chemotherapy finishes). In others, the new treatment is combined with standard chemotherapy in all patients at the start of chemotherapy, and then continues as maintenance monotherapy. The design we propose here is different from these two approaches: preselect patients (based on histology and tumor response), but the assessment of response is made *during* chemotherapy. The new treatment is only given to those with at least stable disease, but is combined with chemotherapy for the remaining cycles, and then as maintenance monotherapy after chemotherapy finishes. The important feature is the *early* assessment of tumor response during standard chemotherapy. This strategy could be applied to patients with either squamous or nonsquamous histology. The proposed approach would be especially useful where patients would not be selected for treatment on the basis of a validated biomarker (e.g., epidermal growth factor receptor [EGFR] mutation). Histology and early response could, in fact, be a surrogate of a predictive biomarker(s) not yet identified.

The JMEN, SATURN, and PARAMOUNT trials are examples of studies that used tumor response to preselect a subgroup of patients, but only after they completed all chemotherapy, and only to investigate maintenance treatment, pemetrexed or erlotinib [2, 5, 6]. In the JMEN study with maintenance pemetrexed, both OS and PFS were improved in nonsquamous patients (OS HR = 0.79, *P* < 0.012; PFS HR = 0.6, *P* < 0.0001), but not in squamous patients (OS HR = 1.07, *P* = 0.68; PFS HR = 1.03, *P* = 0.9) [2]. In the SATURN trial maintenance, erlotinib improved OS (HR = 0.81, *P* = 0.009) and PFS (HR = 0.71, *P* < 0.0001) [5].

If future trials adopting the design we propose here were to confirm the findings, this would have an important influence on finding new targeted treatments, especially where there is no known predictive biomarker. The results of many studies of angiogenesis and targeted treatments in lung cancer have been disappointing because of the lack of an effective biomarker to select patients who can benefit from these agents [7]. Histology and tumor response, as eligibility criteria, are readily available, without the need for collecting and analyzing blood or tumor samples, and validating the biomarkers prospectively, a process which can be expensive, time-consuming, and delay treatment. Selecting patients on the basis of histology and tumor response, instead of biomarkers, could also allow more patients to be randomized in trials of new agents because there might be more patients with these characteristics than those who are biomarker positive. For example, in the EURTAC study, comparing erlotinib with chemotherapy in patients with EGFR activating mutations, 1227 patients were screened but only 174 (14%) were eligible [8].

Several studies show that NSCLC patients have different outcomes according to histology. Patients with nonsquamous tumors have responded better to pemetrexed/cisplatin than gemcitabine/cisplatin (HR = 0.81, *P* = 0.005), compared to squamous tumors (HR = 1.23, *P* = 0.05) [1, 2]. A systematic review of first-line platinum therapy found that cisplatin-based combinations were superior for third-generation regimens among nonsquamous patients (HR = 0.89, 95% CI: 0.81–0.99), but with no clear benefit among squamous patients [9]. Bevacizumab, an angiogenic inhibitor, is currently licensed to treat patients with nonsquamous histology only based on the ECOG 4599 trial. This study demonstrated improved median OS among nonsquamous patients given bevacizumab plus carboplatin/paclitaxel compared with chemotherapy alone (HR = 0.79, *P* = 0.003) although other studies did not show survival benefit [10–12]. SCC patients were excluded from bevacizumab-based trials because of increased life-threatening and fatal pulmonary hemorrhages seen in a phase II study, despite a paradoxical observation of significant cavitating response seen in squamous tumors [13]. It has been postulated that the increased risk of severe pulmonary hemorrhage may be related to the usual central location and propensity for cavitation but another possibility is a significant “super” response.

Other antiangiogenic studies also do not report benefit among SCC. A recent phase III study (MONET) investigating the multikinase tyrosine kinase inhibitor motesanib (AMG 706) plus carboplatin/paclitaxel reported an increase incidence of hemoptysis in SCC patients [14]. In the ESCAPE trial, patients with squamous histology receiving sorafenib plus carboplatin/paclitaxel had an increased incidence of fatal bleeding events and an unexplained increased risk of death (HR = 1.85) [15]. It is not clear why our SCC patients benefited from thalidomide but nonsquamous patients had a poorer survival in contrast to the above antiangiogenesis trials [10, 15, 16]. Apart from inhibiting tumor angiogenesis by interfering with fibroblast growth factor (FGF)/fibroblast growth factor receptor (FGFR) signaling pathway in SCC [17], it is also possible that thalidomide may inhibit the amplified or mutated FGFR oncogenic aberrations often seen in SCC but rarely seen in lung adenocarcinoma [18]. In SCC, amplification of FGFR1 is seen in up to 20% of tumors and our trial design may fortuitously further enrich the FGFR1 positivity rate, thereby allowing thalidomide to work [19, 20].

It is of interest whether thalidomide exerts other antitumor effects beside antiangiogenesis, given the lack of benefit clinical trials using antiangiogenic agents (including bevacizumab) for the treatment of SCC. Translational work on our thalidomide trial, in which we examined vascular endothelial growth factor (VEGF), soluble truncated form of VEGF receptor-2 (sVEGFR-2), interleukin-8, tumor necrosis factor-*α*, basic FGF, and soluble intercellular adhesion molecule-1 in the plasma did not show any association with response to thalidomide [21]. Unfortunately, we did not collect paraffin blocks in our trial to examine FGFR expression or immuno-modulatory markers in SCC in order to study whether they correlate with clinical benefit of thalidomide.

Currently, there is an unmet need for first-line trials in patients with squamous histology. The results from our thalidomide study presented here on PFS were sufficiently statistically significant to warrant a prospective study, especially given that the effect was stronger when focusing only on those who had a complete or partial tumor response. We designed a randomized double-blind placebo-controlled phase II trial of BIBF 1120 (Vargatef ™, Boehringer Ingelheim GmbH, Ingelheim, Germany), an oral triple angiokinase inhibitor that inhibits VEGFR-2, platelet-derived growth factor receptor, and also FGFR, for patients with squamous histology who have at least stable disease after two cycles of gemcitabine/cisplatin (LUME-Lung 3), which is currently ongoing (http://clinicaltrials.gov/ct2/show/NCT01346540) [22]. BIBF 1120 would then be given daily from chemotherapy cycles 3 to 6, and as maintenance. A subgroup analysis would compare the effects between SCC patients with stable disease and complete/partial response. We believe that the timing of BIBF 1120 (i.e., after two cycles of chemotherapy) is important. This enriching strategy will exclude patients who are unlikely to benefit from conventional upfront chemotherapy treatments, and potentially reduce the risks of severe pulmonary hemorrhage due to central cavitations, given that the risks are highest early during treatment. Other studies could explore the new thalidomide analogs based on our findings.

In routine practice, patients are often staged with imaging after two cycles and further chemotherapy is not given to nonresponding patients. Our design of introducing a biological agent only for stable or responding patients after two cycles will therefore complement the current clinical algorithm. In the case of antiangiogenic treatment trials, it also allows exploitation of its tumor vasculature and intratumoral pressure normalizing properties, which can further improve cytotoxic drug delivery for the remaining chemotherapy treatments. This strategy of “enriching” the patient population for additional treatments may also maximize the chance of finding a more successful drug combination and boosting biological trials (where a biomarker has yet to be identified), including those targeting squamous tumors.

In conclusion, our findings suggest that SCC patients who responded or had stable disease after two cycles had improved PFS and OS when thalidomide was given during the remaining chemotherapy cycles and as maintenance. A prospective randomized phase II study is underway to confirm or refute this approach, using a different antiangiogenic agent with FGFR inhibiting property, to investigate the possibility that this is an effective strategy for selecting patients likely to benefit from some novel agents. This enriching strategy should also be investigated in other studies using different antiangiogenic agents and other classes of drugs with no validated biomarkers. We also suggest that researchers of ongoing trials should examine their data in relation to both histology (either nonsquamous or squamous) and early assessment of tumor response, as prespecified subgroup analyses.
